# Adjusting the Balance between Effective Loading and Vector Migration of Macrophage Vehicles to Deliver Nanoparticles

**DOI:** 10.1371/journal.pone.0076024

**Published:** 2013-10-08

**Authors:** Ya-Nan Chang, Haili Guo, Juan Li, Yan Song, Mingyi Zhang, Junjiang Jin, Gengmei Xing, Yuliang Zhao

**Affiliations:** 1 Chinese Academy of Science Key Lab for Biomedical Effects of Nanomaterials and Nanosafety, Institute of High Energy Physics, Chinese Academy of Science, Beijing, China; 2 Chinese Academy of Science Key Lab for Biomedical Effects of Nanomaterials and Nanosafety, National Center for Nanoscience and Technology of China, Beijing, China; University of Sassari, Italy

## Abstract

The nature of macrophage allows the possibility that this cell type could be used as drug delivery system to track therapeutic drug nanoparticles (NPs) in cancer. However, there is no existing research on the regulation between effective loading of NPs and targeted delivery of macrophages. Here, we investigated the important parameters of intracellular NP quantity and the vector migration rate. Macrophage loading capacity was obtained by comparing the uptake quantity of varisized NPs, and the delivery ability of loaded cells was determined by measuring vector migration rates. We observed a positive correlation between the size of NPs and directed macrophage migration. Our findings suggest that the molecular mechanism of migration vector rate regulation involved increased expression levels of colony-stimulating factor-1 (CSF-1) receptor and integrin induced by 100-nm and 500-nm particles. The ability of macrophages uptake to varisized NPs showed the opposite trend, with the increased vector rate of cell migration influenced by NPs. We are able to demonstrate the important balance between effective macrophage loading and targeted delivery. By adjusting the balance parameters, it will be possible to utilize NPs in macrophage-mediated disease diagnosis and therapy.

## Introduction

Macrophages are versatile cells that play critical roles in both pathologic and physiologic responses [1]. As their name implies, macrophages can phagocytose and dispose of worn-out cells and other debris, and migrate into areas affected by inflamation or infection. Nanoparticles (NPs) used in macrophage-mediated disease treatments could be an extremely useful tool [2]. Drugs, peptides and nucleic acids have been combined with polymers and lipids to generate NPs that have the ability to interact with and be internalized by cells [3], [4], especially macrophages.

Macrophages are recruited to migrate into many types of tumor tissue and appear to be directed involved in tumor progression and metastasis [5], [6]. Two lines of evidences derived from clinical and epidemiological studies indicate that a high density of macrophages in tumor tissue correlates with poor prognosis. Therefore, macrophages represent an important means of cancer diagnosis and could also serve as a way to target treatments to cancerous tissues [7]. Some studies have demonstrated that macrophages can serve as vehicles to deliver therapeutic drugs or fluorescence agents for diagnosis [8]. Work by Kingsley and colleagues supported the idea that macrophage-based drug delivery systems could be used to administer therapeutic NPs in human disease [9]. However, the obstacles to realize these goals include cell uptake of drugs and appropriate monocyte trafficking to tumor tissues and disease sites. To achieve these goals, it is necessary to understand the kinetics of NP uptake and their distribution in macrophages.

It has been widely demonstrated that macrophages are phagocytic cells that can serve as useful nanosized-drug carriers [10]. NPs can be altered to exploit these characteristics. For instance, liposome-protamine-DNA (LPD) NPs coated with mannan enhance antitumor activity because mannose receptors are expressed on the surface of macrophages [11]. Colloidal gold NPs coated with human and rat plasma fibronectin are rapidly bound and endocytosed by macrophages [12]. Polyanionic macromolecules and superparamagnetic iron oxide NPs are known to bind to the surface of macrophages and are subsequently internalized [13], [14]. Size appears to be an important factor in these events; some studies demonstrated a direct relationship between NP size and macrophage uptake [15], [16]. Jiang *et al*. showed that cells interact with specifically sized NPs at the molecular level and further demonstrated that binding and activation of cellular membrane receptors and subsequent protein expression were strongly dependent on NP size [17]. Interestingly, the ability of macrophages to migrate to tumor sites involves the expression of specific proteins, e.g., colony stimulating factor-1 (CSF-1) receptor [18] and integrin [19], [20]. We hypothesized that an appropriate balance in macrophage uptake of NPs and trafficking to tumor tissues can be achieved by using specific sized NPs.

To achieve such a balance, it is necessary to understand macrophage migration behavior, which is governed by internal NP size. Nel’s laboratory showed that 60-nm cationic NPs were highly toxic to macrophages, and it is likely that cell-specific uptake mechanisms and pathways influence sensitivity or resistance to cationic particle toxicity [21]. Many studies have demonstrated that NPs induce inflammatory cell recruitment to the lung following particle deposition *in vivo* [22]. To avoid toxic effects of NPs, one group employed different sized, negatively charged, fluorescent-labeled COOH-polystyrene (PS) [23]. To demonstrate that NP size actively affects the uptake and the subsequent effects on cellular migration, we assessed macrophage uptake of varisized NPs and measured the impact of NP size on the ability of macrophages to migrate to cancer cells *in vitro*. Our results, taken together, demonstrated NPs with 100-nm diameters increased the vector migration rate and were more effectively taken up by macrophages. Our findings suggest a new line for macrophage-mediated disease detection and treatment by employing appropriately sized NPs.

## Materials and Methods

### Cell Culture

RAW 264.7 (Mouse leukemic monocyte macrophage cell line) and MDA-MB-231 (human breast cancer cell line) cells were cultured in 50-cm^3^ culture flasks in 10 ml Dulbecco’s modified Eagle’s medium (DMEM)/F-12 1:1 (Hyclone Laboratories, Logan, UT, US) with 10% fetal bovine serum (FBS, Hyclone Laboratories). To imitate the tumor microenvironment, RAW264.7 cells were cultured in medium with CSF-1 (36 ng/ml, Sigma, St. Louis, MO, USA) for 4 h in each group prior to the experiments. Media were changed every second day and cells were harvested with trypsin-EDTA (phosphate-buffered saline [PBS] containing 0.53 mmol/L EDTA and 0.05% trypsin). Cells were incubated at 37°C in 5% CO_2_ in humidified air.

### Immunofluorescence

After about 2×10^5^ RAW 264.7 cells were cultured with the same number in each plate, NPs (latex beads carboxylate-modified polystyrene, with diameters of 30, 50, 100, and 500 nm, Sigma) were added into the culture medium (final concentration, 25 ng/ml). The treatment groups were incubated with different NPs for 4 h, and the control group contained only culture medium and CSF-1. All cells were fixed in 4% formaldehyde (Sigma) in PBS for 30 min, and treated with 0.1% Triton X-100 (AMRESCO LLC, Solon, OH, USA) and blocked with 10% goat serum in PBS. CSF-1 receptor was visualised with 1∶75 rabbit anti-CSF-1 receptor α. Cells were then washed thoroughly and incubated with TRITC anti-rabbit antibodies (Zhongshan Goldenbridge Biotechnology, Beijing, China). Cell tubulin was visualized with phalloidin (Life Technologies). Images of cells were acquired with a laser confocal scanning microscope (PerkinElmer Ultra VIEW VoX, PerkinElmer, Waltham, MA, USA: with a Nikon TI-E inverted microscope, Nikon, Tokyo, Japan) equipped with a 100×oil immersion lens. The quantity of intracellular NP green fluorescence was analysed with Volocity software (PerkinElmer).

### Cell Migration Assay (Boyden Chamber Migration Assay)

Migration assays were carried out in a Boyden chamber with a microporous membrane containing 8-µm pores (Millipore, Billerica, MA, USA). Two groups of experiments were designed; in the control group, 100% FBS was in the lower chamber, in the experimental group, MDA-MB-231 cells (0.2×10^5^/ml) were seeded in 300 µl 10% FBS-DMEM into the lower wells. After culturing for 12 h, 2×10^5^/ml RAW 264.7 cells treated with various sizes of NPs (30, 50, 100 or 500 nm) at a treatment concentration of 25ng/ml were loaded into the upper wells.Cells were pretreated with CSF-1 (36 ng/ml) for 4 h prior to migration chamber loading. After 6 h incubation (37°C, 5% CO_2_), non-migrated cells were removed and washed three times in PBS (pH = 7.2). Migration was measured by counting cells on the underside of the membrane after 4% paraformaldehyde fixation and Hoechst staining (0.5 µg/mL, Hoechst 33342, Life Technologies). The number of migrated cells was determined by inverted fluorescence microscopy (Olympus Corporation, Tokyo, Japan) and assays were performed in triplicate.

### Cell Tracking Assays

To quantitatively analyse the effect of varisized NPs on RAW 264.7 cell motility, we generated a polydimethylsiloxane (PDMS) cell culture setup, so that two kinds of cells shared substrate and culture medium, and grew on the same plate without direct contact. This setup created a 300-µm separation between the two cell types, and each of which grew in a 500 µm-wide trip. This allowed cytokines to secrete to the other cell type, simulating the *in vivo* environment. After treatment with or without 30-nm, 50-nm, 100-nm, or 500-nm nanospheres for 4 h, 5×10^6^/ml RAW 264.7 cells were seeded into a PDMS groove in 2.5-cm tissue culture plastic dishes, and the other groove was filled with 1.5×10^6^/ml MDA-MB-231 cells. After 10 h, the PDMS cover was removed, the medium was replaced with media containing CSF-1 (36 ng/ml), cell migration was monitored in 10-min intervals for 24 h by time-lapse video microscopy system using Volocity Quantitation software, and analyzed using Openlab software (Improvision, Coventry, UK). We calculated 60 cells for each subtype (20 cells per experiment, for three separate experiments) using a laser scanning confocal microscope cell real-time imaging system with the same gain and offset settings for all sections. Analysis of cell speed and directionality was carried out using SPSS 16.0 software.

To provide an indication of macrophage trajectory, the directional persistence was calculated. The persistence (T) is trajectory rate, (V) is vector rate of final displacement of a cell from its origin during the time-lapse film, (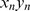) is the coordinate position of cells, and (t) is the total time: 
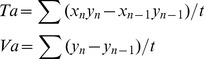



### Quantitative Real-time Polymerase Chain Reaction (RT-PCR) and Western Blotting

For the evaluation of mRNA and protein expression levels of cytokine (CSF-1), the total RNA transcripts and total protein translations from RAW 264.7 cells were prepared as described for immunofluorescence, and after incubation, cells were treated with TRIzol reagent (Life Technologies) and Lysis Buffer, according to the manufacturer’s protocol. However, cells prepared in experimental groups for β1-integrin expression were incubated in varisized NP culture medium (2.5 µg/ml) for 4 h. The total RNA and protein solutions were stored at −80°C until use. Semi-quantitative RT reactions were conducted as reported previously [24]. The primers used in this experiment were as follows: CSF-1 receptor: forward, TGG TGC ACC CCT AGT TCT CT; reverse, GGC CAC TCC TGT GAG CTT AG. Integrin β1: forward, AGT GAA TGG GAA CAA CGA GGT C; reverse, CAA TTC CAG CAA CCA CAC CA. GAPDH: forward, AGG CCG GTG CTG AGT ATG TC; reverse, TGC CTG CTT CAC CAC CTT CT. Western blotting procedures were as described by Kindwall-Keller *et al.* [25]. The Anti-CSF-1 receptor and integrin β1 antibody were purchased from Santa Cruz Biotechnology (Santa Cruz, CA, USA) and abcam (Cambridge, UK), respectively.

### Fluorescein-activated Cell Sorter (FACS) Analysis

Cell preparation was performed as described above, and cells were washed in PBS, fixed in 4% formaldehyde in PBS for 30 min, and then treated with anti-mouse CSF receptor α at the recommended dilution (1∶200), at 4°C, overnight. Goat rhodamine-conjugated anti-rabbit IgG (Zhongshan Goldenbridge, Beijing, China) was used as a secondary antibody and incubated for 3 h before detection and analysis by BD FACS Calibur (BD Biosciences, New Jersey, USA).

### Statistical Analysis

Statistical analysis was performed with SPSS 16.0 software (SPSS Inc., Chicago, IL, USA). Data are expressed as means ± standard deviation (SD). One-way analysis of variance (ANOVA) was carried out to compare the differences of means when three or more groups were assessed. For cell migration assays, we performed two-sample t-tests assuming equal variance, and *p* values were calculated with two-tailed tests. *p*<0.05 was considered statistically significant.

## Results

### NPs Affect Macrophage Migration to Cancer Cells

Macrophage chemotaxis can be regulated by growth factors in tumor tissue [26], [27]. Microarray analysis and functional testing previously revealed that CSF-1, which was excreted by cancer cell, is the major chemoattractant for RAW 264.7 macrophages migration to cancer cell [28].

**Figure 1:**
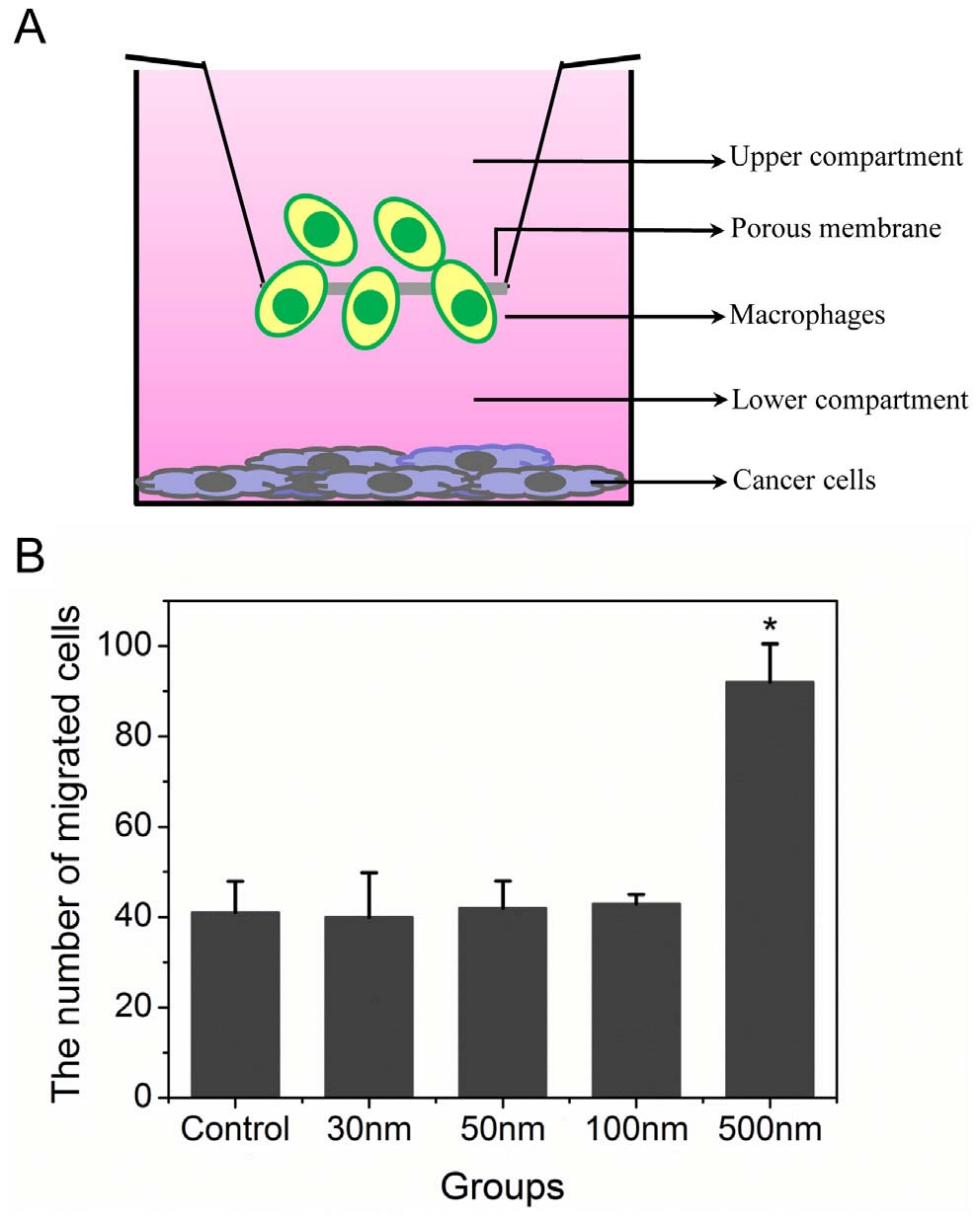
RAW 264.7 cells migration with or without uptake of varisized NPs. A) Schematic description of the Boyden chamber assay. RAW 264.7 cells were added to the upper chamber of the Boyden chamber and allowed to migrate through the porous membrane into the lower chamber containing medium alone or medium with MDA-MB231. Cells were pretreated with culture medium or varisized NPs. B) Migrated cells on the lower side of the membrane were stained with Hoechst 33342 and counted under a fluorescence microscope using a ×20 objective. The histogram illustrates the number of migrated cells in different treatment. *p<0.05 compared with control (Error bar is SEM).

To investigate the cancer cell-induced chemotactic capability to regulate macrophage migration, both cell types were co-cultured in modified boyden chamber. MDA-MB-231 cells were added to the lower compartment, and RAW 264.7 cells that were pretreated with NPs were seeded in the upper chamber (Fig. 1A). The cellular density of control cells was 41.5±6.95, and values of 40±9.82, 42±5.99, 43.5±2.03 and 92±8.48 corresponded to treatment with NPs of 30, 50, 100 and 500 nm, respectively (Fig. 1B). Compared to the control condition, most sizes of NPs (30, 50 and 100 nm) did not significantly alter macrophage density. However, more 500-nm-treated macrophages migrated through the porous membrane compared with the other groups (*p*<0.05).

**Figure 2:**
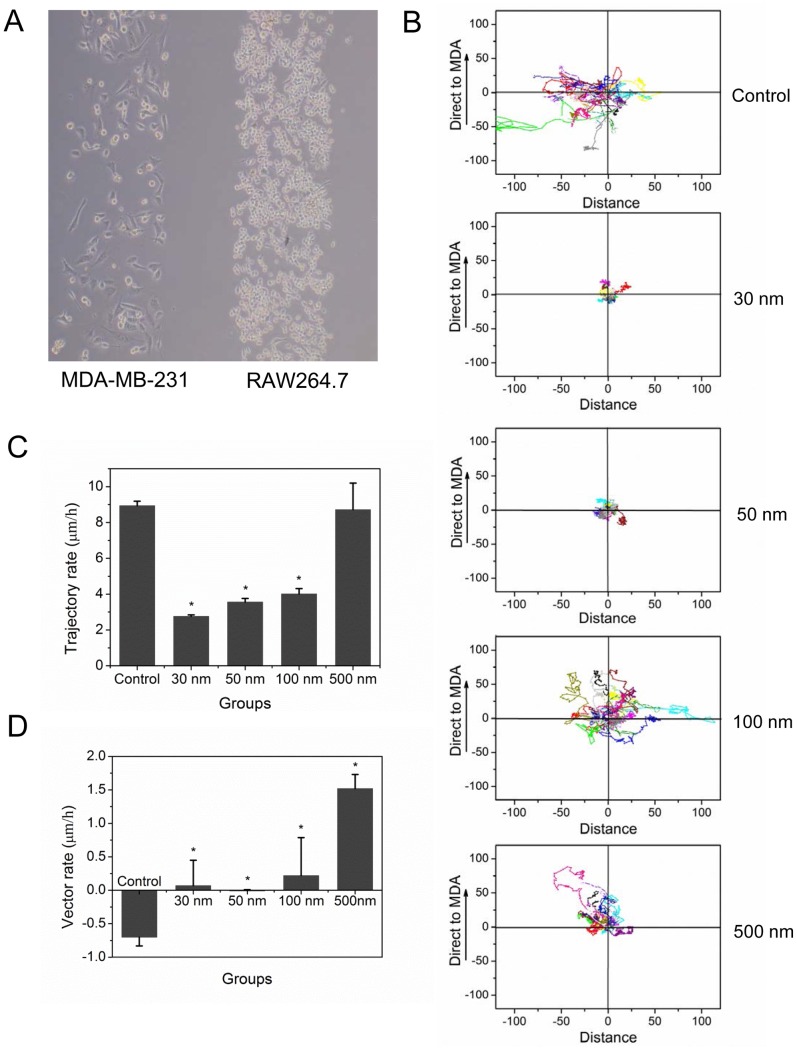
RAW 264.7 cells vector migration ability. A) Schematic of the optical microscope with microfluid technology for assessing macrophage vector migration ability. B) RAW264.7 cell mobility changed after NP uptake. Cell movement was followed by live confocal microscopy for 24 h. The origin of each cell is at the intersection of the x- and y-axis, 20 cells were assessed per experiment (performed in triplicate). Cell migration over a defined area was determined in fresh media in the control group vs. pre-treatment with 30-, 50-, 100-, and 500-nm NPs, respectively. The results were analyzed, and the histograms illustrate the trajectory C) and vector D) rate of RAW264.7 cells following different pre-treatments. *p<0.05 compared with control (Error bar is SEM).

To accurately measure the trajectories of pretreated macrophages, we employed microfluid technology and real-time confocal imaging. We established a co-culture system for RAWs and MDA-MB-231 (Fig. 2A), in which RAW 264.7 cells were pre-treated with fluorescent NPs with various diameters before we tested their ability to migrate toward cancer cells. Cell movement was monitored under the confocal microscope for 24 h, and the results were analyzed with Openlab software. The line-map of each pretreated RAW 264.7 cells trajectory is shown in Figure 2B. The starting point of cell movement was regarded as the origin of the coordinate axis, and the y-axis corresponds to RAW 264.7 cells moving toward MDA-MB-231 cells in the lower chamber. The range of vertical and horizontal coordinates of all maps is from -120 to 120 (µm).

The migration trajectories of RAW 264.7 treated by NPs (Fig. 2B) showed that 30- and 50-nm NPs sharply reduced macrophage migration trajectory rate (T) compared to the control group. However, 100- and 500-nm NPs did not have the same effect. Moreover, 500-nm NPs were more likely to appear in the area above the x-axis (Fig. 2B). These observations were confirmed with precise mathematical calculations. The statistical results (Fig. 2C) showed that the T values were 8.98±0.25, 2.76±0.08, 3.56±0.19, 4.01±0.29, and 8.72±1.48 for the control, 30-, 50-, 100-, and 500-nm NP groups. The results demonstrated that the smaller-sized NPs significantly depress macrophage migration, whereas the larger-sized (500-nm) NPs did not. The vector rates (V) of macrophages containing NPs are shown in Figure 2D. The V values were −0.71±0.13, 0.07±0.38, −0.01±0.02, 0.22±0.56, and 1.52±0.21 m/h, corresponding to the control, 30-, 50-, 100-, and 500-nm groups, respectively (Fig. 2D). The results show that macrophage migration rates (both T and V) increased when the internal NP diameter increased from 30 to 500 nm, which suggested that we could regulate macrophage migration capability by changing intracellular NP diameter.

### Internal NPs Affect Macrophage CSF-1 Receptor Expression

**Figure 3:**
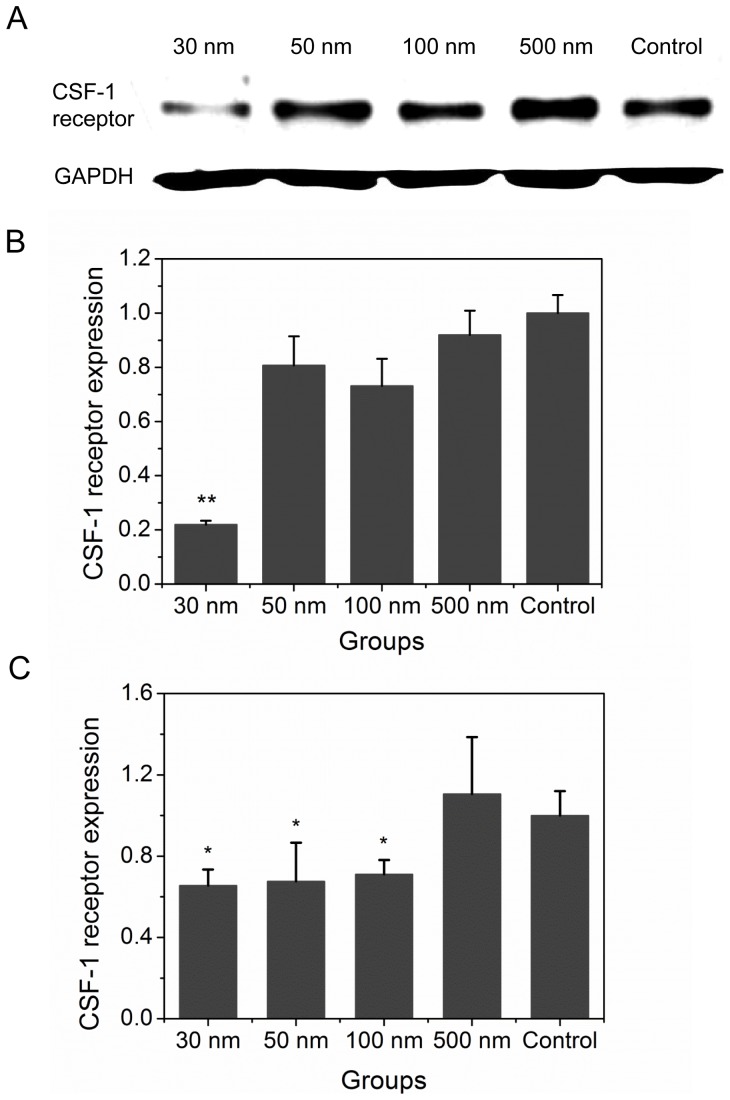
CSF-1 receptor expression after RAW 264.7 uptake of varisized NPs. A) Western blot assay of CSF-1 receptor protein expression. GAPDH served as loading control. B) Histogram showing the results of real-time fluorescent quantitative PCR (Q-PCR) assay of CSF-1 receptor gene expression. *p<0.05 and **p<0.01 compared with control (Error bar is SEM).

**Figure 4:**
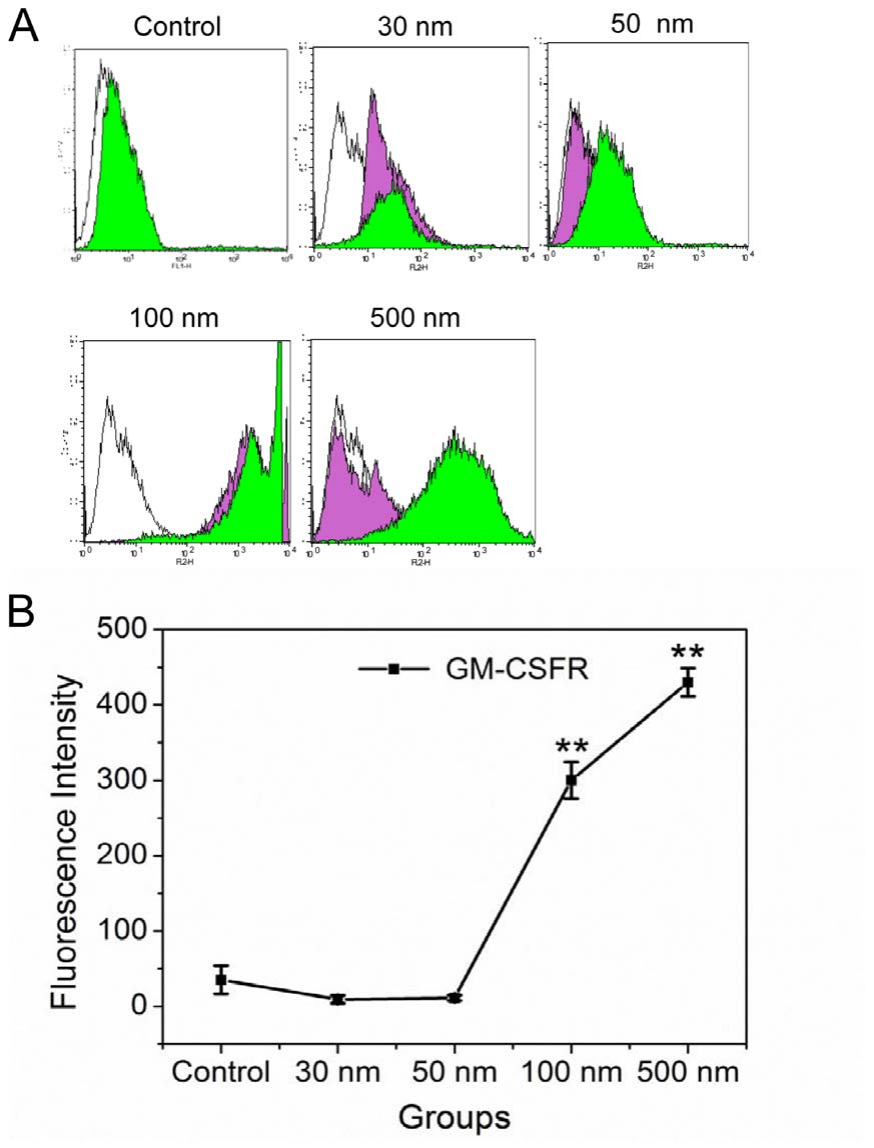
Flow cytometry assessment of CSF-1 receptor protein in RAW 264.7 cells. A) CSF-1 receptor expression on RAW 264.7 cell surface; the green histograms represent the fluorescence intensity of cells carrying NPs after immunofluorescence staining, white histograms depict control group cells without NPs, and the violet histograms indicate the fluorescence intensity of cells with only NPs. B) Line graph showing CSF-1 receptor expression in different pre-treated RAWs. **p<0.01 compared with control (Error bar is SEM).

To explore the mechanism of how NPs influence cell vector migration, we examined CSF-1 receptor expression in RAW 264.7 cells by Q-PCR and western blotting. As shown in Fig. 3, the larger-sized NPs induced the higher levels of CSF-1 receptor mRNA and protein expression. Many studies have pointed out that CSF-1 receptor on the surface of macrophages guide macrophage vector migration to cancer cells [29], so we quantified CSF-1 receptor expression in RAW 264.7 cells with flow cytometry (Fig. 4). CSF-1 receptors were labeled with a primary antibody and a red fluorescence secondary antibody. The white, violet, and green peaks depict the negative control, the fluorescence intensity of cells with only NPs, and the fluorescence intensity of CSF-1 receptor, respectively. The results suggest that RAW264.7 cells pre-treated with 500-nm NPs and CSF-1 expressed higher levels of receptors than 100-, 50-, and 30-nm NP-treated cells, as well as control cells, in that order (Fig. 4B).

### Internal NPs Affect Macrophage Migration via Regulation of Integrin Expression and Function

**Figure 5:**
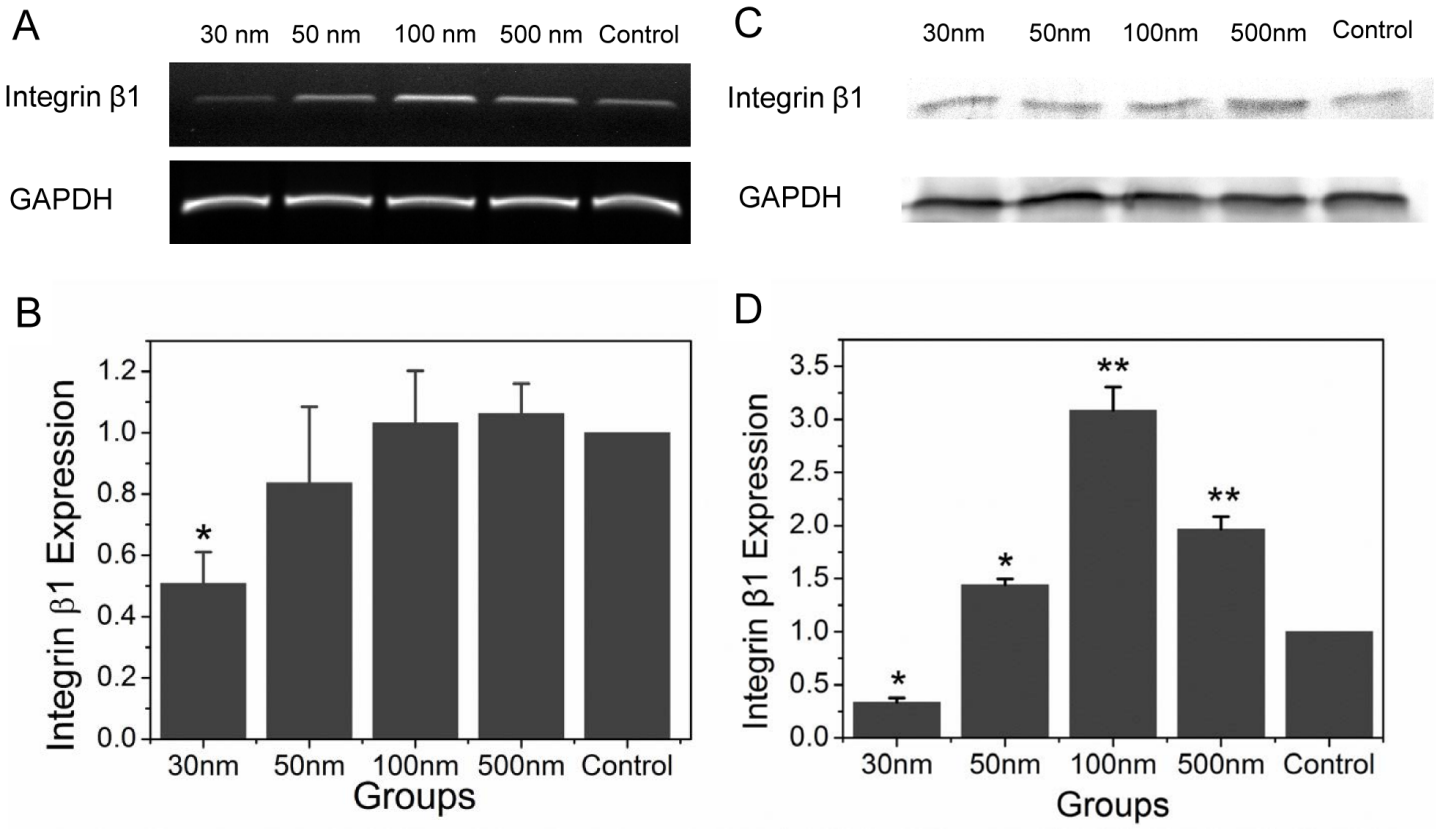
Integrin β1 expression following RAW 264.7 cells uptake of varisized NPs. A) Integrin β1 mRNA levels were determined by semi-quantitative PCR (grayscale analysis is shown in B), and C) protein expression was analyzed by western blotting (grayscale analysis is shown in B). GAPDH served as a loading control. *p<0.05, **p<0.01 compared with control (Error bar is SEM).

Integrins can bind to specific cell-surface, extracellular matrix, or soluble protein ligands and thus play a central role in cell adhesion and migration. They provide a transmembranous link for bidirectional mechanical force transduction across the plasma membrane and can affect the spatial dynamics of cytoskeletal organization, leading to directional cell movement [30]. We assessed integrin β1 mRNA expression by semi-quantitative PCR (Fig. 5A and B) and analyzed protein expression by western blotting (Fig. 5C and D). Compared to control, all NPs (except 30 nm) induced integrin mRNA expression, and 100-nm NPs had the strongest effect. The effect of 30-nm NPs was significant compared to the control group, and these changes were more pronounced in the 50-, 100-, and 500-nm NP groups. Western blotting results confirmed these findings. Collectively, these results support the hypothesis that internalized NPs affect macrophage integrin expression.

### Size-dependent Macrophage Uptake and Morphological Changes

**Figure 6:**
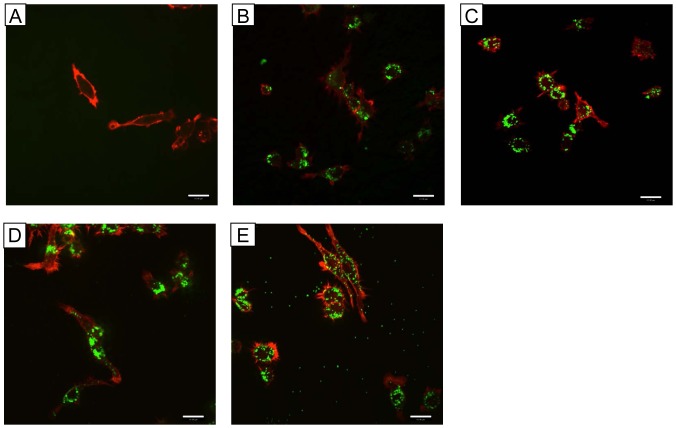
High-resolution imaging with rhodamine phalloidin-labeled actin shows NP-containing cell morphological. RAW incubated A) control, B) 30 nm, C) 50 nm, D) 100 nm, and E) 500 nm NPs at 37°C for 4 h. The cells were fixed and labeled with phalloidin. Two-color immunofluorescence confocal images were obtained for NPs (green) and G-actin (red).

Cell morphology and migration ability are closely related in macrophages, and integrin binding to extracellular ligands induces cell morphology changes. We incubated RAW 264.7 cells with NPs and cytokine for 4 h before labeling them with rhodamine phalloidin. The cells in the control group (Fig. 6A) showed one or more rich lamellae and a tail. Conversely, cells incubated with 30- (Fig. 6B) or 50-nm (Fig. 6C) particles exhibited dendritic features with more than two protrusions. Macrophages treated with 100- (Fig. 6D) or 500-nm (Fig. 6E) NPs appeared similar to the control group. Directional migration is initiated by extracellular cues, such as gradients of growth factors or chemokines.

**Figure 7:**
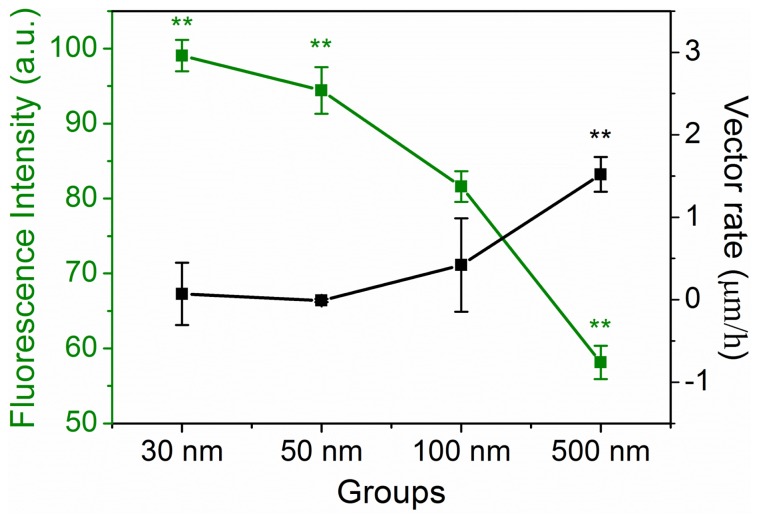
The relationship between effective NP loading and RAW 264.7 cell vector migration. Fluorescence intensity statistics for RAW264.7 cells treated with four varisized intracellular NPs (green line). Vector rate of RAW264.7 cells for different pre-treatment conditions (black line). *p<0.05, **p<0.01 compared with 100-nm NPs treated group (Error bar is SEM).

To evaluate the ability of macrophages to uptake NPs, we cultured RAW 264.7 cells, added varisized NPs (30, 50, 100, and 500 nm) to the culture medium, and then assesses intracellular NP quantity with a fluorescent microscope. After 1 h, RAW 264.7 cells exhibited diverse NP uptake kinetics (see Fig. S1). We observed that 30-nm green fluorescent NPs rapidly entered and distributed throughout the cytosol. Notably, 50-, 100-, and 500-nm NPs first gathered on the cell membrane before crossing the lipid membrane and entering the cytosol. NP fluorescence quantity was considered as a surrogate of the number of intracellular NPs, and we analyzed green fluorescence in every cell. The graph (green line, Fig. 7) clearly demonstrates that the number of NPs entering the cell decreased as NP size increased. The result indicates that NP size was negatively correlated with NP uptake rate, and smaller NPs are more effectively loaded in macrophages.

## Discussion

In tumor microenviroments, macrophages transform into migratory hematopoietic cells and may be useful as therapeutic and diagnostic targets [6]. Ikehara *et al*. established the utility of intraperitoneally injected macrophages as a novel drug delivery system for the control of cancer metastasis to milky spots, which are considered an initial location for disseminated cancer cells to develop into solid tumors [8]. Zeisberger *et al*. demonstrated that clodronate encapsulated bisphosphonate clodronate into liposomes with a mean diameter of 135±55 nm, mediated macrophage depletion, and inhibited tumor growth [31]. As particulate foreign bodies, NP systems are capable of inducing a host response, such as inflammatory cytokines secretion. Chellat *et al.* [3] found that matrix metalloproteinase-9 (MMP-9) secretion by THP-1-macrophages increased over time during a 24-h incubation with NPs.

Malignant cancer cells release CSF-1, which induces macrophages to express the CSF-1 receptor [18]. In order to imitate the tumor microenvironment, we introduced the chemotactic factor CSF-1 into our experimental protocol. Compared with the control group, internalized NPs induce macrophages to express CSF-1 receptor, and this effect was more pronounced in cells treated with larger NPs. The flow cytometry results verified those of western blotting and showed that 100- and 500-nm NPs induced the highest levels of CSF-1 receptor expression (Figs. 3 and 4). CSF-1 receptor expression on the macrophage surface was not significantly different among the control and 30- and 50-nm NP-treated groups.

Macrophages with high cell-surface CSF-1 receptor expression influence the high vector rate of directed migration toward cancer cells. We calculated the vector rate of pretreated macrophage migration to cancer cells and observed a size effect; internalized NPs from 30 to 500 increased vector rate, and larger sizes had stronger effects. However, the migration trajectory rate results suggest that 30- and 50-nm NPs sharply depress macrophage migration, whereas 100- and 500-nm NPs did not significantly decrease macrophage migration. It is noteworthy that control macrophages exhibited more locomotivity and a higher trajectory rate value than cells treated with 30- and 50-nm NPs. In figure 2D, the vector rate was negative in the control group, which suggested that macrophages were able to migrate but were unable to direct migration toward the cancer cells. However, the locomotivity of macrophages was strongly related to NP size. Larger NPs increased both the vector and trajectory rates of macrophage migration. Smaller NPs (30 and 50 nm) restrained macrophage migration by decreasing both vector and trajectory rates.

Cell migration begins with the cellular response to an external signal that leads to polarization and the protrusion extension in the direction of the signal [32]. Cells with less branched, ramified morphological phenotypes showed stronger migration [33], whereas cells with long and stable protrusions indicated the direction of migration. To migrate, a cell must acquire spatial asymmetry that enables them to transduce intracellularly generated forces into net translocation [34]. One manifestation of such asymmetry is polarized morphology [35]. Additional molecular rearrangements, such as redistribution of chemosensory signaling receptors, integrin adhesion receptors, and cytoskeletal components also facilitate migration [34]. Cell polarization in response to migratory stimuli is almost always coupled with local actin polymerization [36]. In our experiment, rhodamine phalloidin was used to visualize cellular morphology to allow us to assess actin polymerization location. The cells in the control group exhibited one or more rich lamellae and tails. Cells incubated with 30- and 50-nm NPs had dendritic features with more than two protrusions. Cells with less branching or ramified morphological phenotypes showed stronger migration, and those forming long and stable protrusions indicated the direction of migration [33]. Both 100- and 500-nm NPs induced actin polymerization and less branching. These morphogenetic processes suggested that larger NPs (100 and 500 nm) more strongly activated macrophage migration compared to 30- and 50-nm NPs.

In general, the coordination of adhesion, signaling, and cytoskeletal spatial dynamics organization result in directional cell movement [35]. Integrins are heterodimeric adhesion receptors formed by non-covalent interactions between α and β subunits that play a central role in this process [37]. We assayed integrin β1 expression in NP-pretreated macrophages and reached the same conclusion as for the CSF-1 receptor experiments. Compared to the control group, NPs with diameters larger than 30 nm were able to induce greater integrin mRNA expression, with 100-nm NPs having the strongest effect. Our results suggest that the internalized 100- and 500-nm NPs induce macrophage polarity by regulating integrin expression.

We also quantified intracellular fluorescently labeled NPs after 4 h and found that smaller (30 nm) NPs have the greatest ability to enter cells. Obviously, larger NPs required more time to enter cells. Notably, NP size had the opposite effect on cell migration; larger NPs were associated with an increased vector rate of cell migration. This is important because macrophages need to uptake large quantities of drugs for this method to be effective for disease diagnosis and therapy. As shown in Figure 7, we tried to find a possible relationship between NP quantity (Q) and the vector rate (V) of macrophage migration. V/Q was regarded as an important parameter to evaluate the drug (NPs) delivery system of macrophages for disease therapy and diagnosis. We found that particles with diameters of about 100 nm would best achieve the balance between high loading capacity and effective target delivery.

## Conclusions

Many studies have indicated that macrophage-based drug delivery systems may be used to deliver therapeutic NPs to sites of human disease [13], [38], [39]. However, it is difficult to achieve both a large drug loading quantity and appropriate monocyte trafficking to target sites. We performed experiments to assess the effective loading and directed delivery of drugs and found that macrophage uptake and NP size both influenced macrophage migration toward cancer cells. Notably, NPs with 100-nm diameters increased the vector migration rate and were more effectively taken up by macrophages. Our results further suggest that smaller NPs (30 and 50 nm) can restrain migration, thereby depleting macrophage density in tumor tissue. Collectively, our findings underscore the importance of employing appropriately sized NPs and may open up a new avenue for macrophage-mediated disease detection and treatment.

## Supporting Information

Figure S1 **Live confocal microscopy imaging of RAW264.7 cells incubated with four sized NPs on different time.** One-hour-phagocytosis-movie screenshot, the three kinds of nanoparticles except 30-nm one, accomplished the process that one particle entered into the cell. 30-nm (A, E); 50-nm took (B, F); 100-nm (C, G) and 500-nm (D, H). The scale bar is 10 µm.
(TIF)
