# Supplementary figures and images for: Adjusting the Balance between Effective Loading and Vector Migration of Macrophage Vehicles to Deliver Nanoparticles
Source: PLoS One. 2013 Oct 8;8(10):e76024. doi: 10.1371/journal.pone.0076024 (PMC3792996; doi:10.1371/journal.pone.0076024)

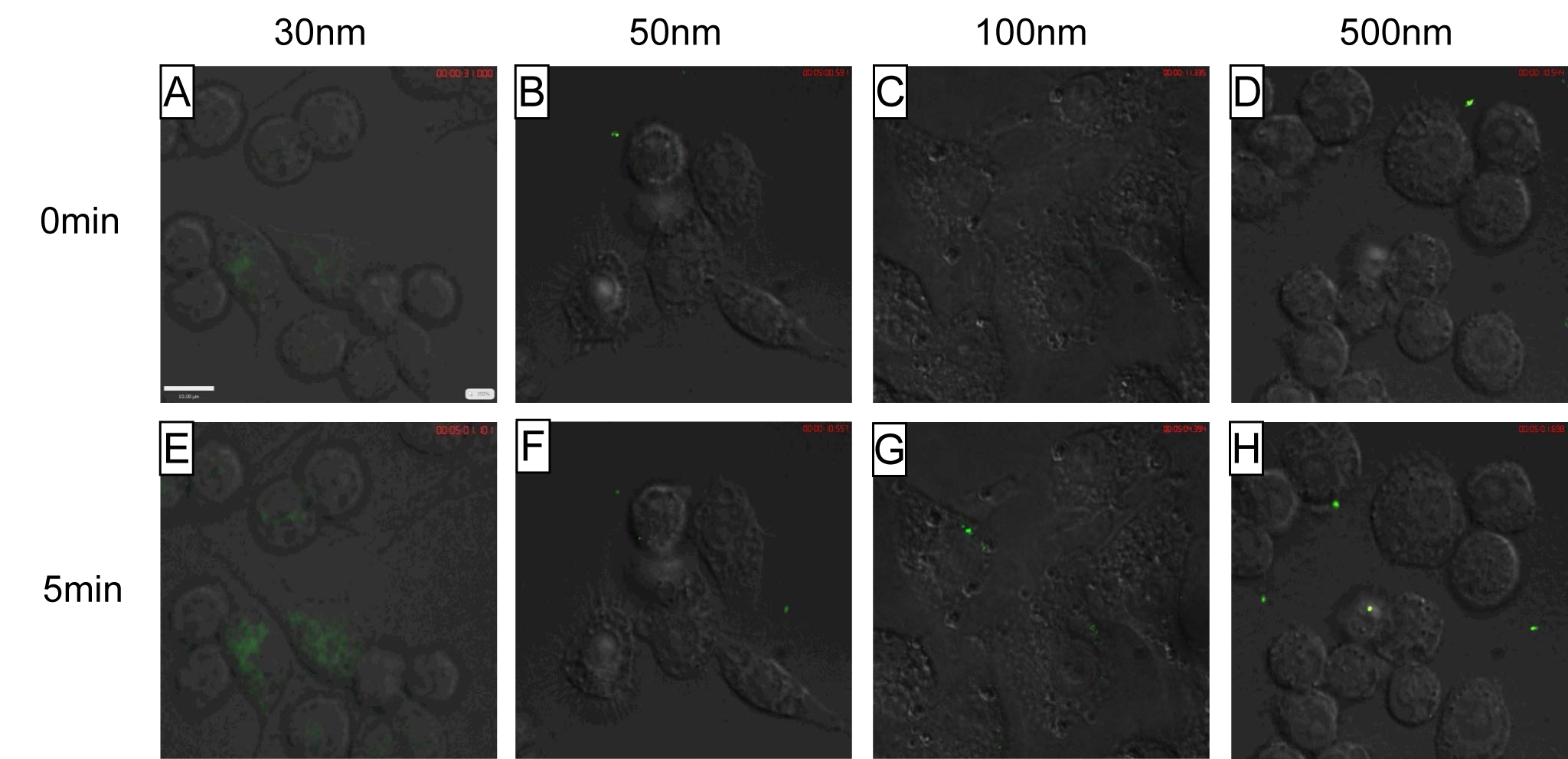

Supplement: Figure S1 — Live confocal microscopy imaging of RAW264.7 cells incubated with four sized NPs on different time. One-hour-phagocytosis-movie screenshot, the three kinds of nanoparticles except 30-nm one, accomplished the process that one particle entered into the cell. 30-nm (A, E); 50-nm took (B, F); 100-nm (C, G) and 500-nm (D, H). The scale bar is 10 µm. (TIF) [file pone.0076024.s001.tif]
